# Dynamic atlas of immune cells reveals multiple functional features of macrophages associated with progression of pulmonary fibrosis

**DOI:** 10.3389/fimmu.2023.1230266

**Published:** 2023-09-13

**Authors:** Jiaoyan Lv, Haoxiang Gao, Jie Ma, Jiachen Liu, Yujie Tian, Chunyuan Yang, Mansheng Li, Yue Zhao, Zhimin Li, Xuegong Zhang, Yunping Zhu, Jianhong Zhang, Li Wu

**Affiliations:** ^1^ Institute for Immunology, Tsinghua-Peking Joint Centre for Life Sciences, School of Medicine, Tsinghua University, Beijing, China; ^2^ Department of Automation, Ministry of Education (MOE) Key Laboratory of Bioinformatics, Bioinformatics Division and Centre for Synthetic & Systems Biology, BNRist, Tsinghua University, Beijing, China; ^3^ State Key Laboratory of Proteomics, Beijing Proteome Research Center, National Center for Protein Sciences (Beijing), Beijing Institute of Lifeomics, Beijing, China; ^4^ Annoroad Gene Technology (Beijing) Co., Ltd., Beijing, China; ^5^ School of Life Sciences, Tsinghua University, Beijing, China; ^6^ Beijing Key Laboratory for Immunological Research on Chronic Diseases, Beijing, China

**Keywords:** macrophages, single-cell RNA sequencing, pulmonary fibrosis, dynamic atlas, pathogenesis

## Abstract

Idiopathic pulmonary fibrosis (IPF) is a chronic interstitial lung disease with a high mortality rate and unclarified aetiology. Immune response is elaborately regulated during the progression of IPF, but immune cells subsets are complicated which has not been detailed described during IPF progression. Therefore, in the current study, we sought to investigate the role of immune regulation by elaborately characterize the heterogeneous of immune cells during the progression of IPF. To this end, we performed single-cell profiling of lung immune cells isolated from four stages of bleomycin-induced pulmonary fibrosis—a classical mouse model that mimics human IPF. The results revealed distinct components of immune cells in different phases of pulmonary fibrosis and close communication between macrophages and other immune cells along with pulmonary fibrosis progression. Enriched signals of SPP1, CCL5 and CXCL2 were found between macrophages and other immune cells. The more detailed definition of the subpopulations of macrophages defined alveolar macrophages (AMs) and monocyte-derived macrophages (mo-Macs)—the two major types of primary lung macrophages—exhibited the highest heterogeneity and dynamic changes in expression of profibrotic genes during disease progression. Our analysis suggested that *Gpnmb* and *Trem2* were both upregulated in macrophages and may play important roles in pulmonary fibrosis progression. Additionally, the metabolic status of AMs and mo-Macs varied with disease progression. In line with the published data on human IPF, macrophages in the mouse model shared some features regarding gene expression and metabolic status with that of macrophages in IPF patients. Our study provides new insights into the pathological features of profibrotic macrophages in the lung that will facilitate the identification of new targets for disease intervention and treatment of IPF.

## 1 Introduction

Idiopathic pulmonary fibrosis (IPF) is the most common form of interstitial lung disease, and its prevalence is increasing worldwide (1). The pathological characteristics of IPF include abnormal activation of myofibroblasts and excess deposition of collagen in the lung, subsequently leading to respiratory failure. However, due to the complexity of the pathogenetic process of IPF and the uncertainty of patient aetiology, IPF is clinically refractory (2). Although two drugs, pirfenidone and nintedanib, have shown the ability to slow down disease progression, neither medication improves or stabilizes lung function (3). A more comprehensive understanding of the molecular and cellular mechanisms of pulmonary fibrosis is required to develop new therapeutic strategies.

Immune cells actively participate in pulmonary fibrosis progression. In particular, lung macrophages, which typically comprise alveolar macrophages (AMs), monocyte-derived macrophages (mo-Macs), and interstitial macrophages (IMs), are crucial for tissue damage, repair, and fibrosis (4). Indeed, several studies have reported the specialised roles of macrophages in tissue repair and fibrosis (5–7). It is believed that macrophages produce large amounts of growth factors to promote myofibroblast proliferation and subsequent tissue repair and remodelling (8, 9). The emergence of single-cell RNA sequencing enables the profiling of thousands of individual cells and further helps to elucidate the disease mechanism. Also, understanding the heterogeneity of macrophages could reveal the potential therapeutic biotarget. Reyfman et al. applied single-cell RNA sequencing (scRNA-seq) and highlighted the primary profibrotic functional population of macrophages in human fibrotic lungs (10). Nevertheless, macrophage subpopulations remain poorly characterised. The unavailability of human samples at different time points also limits the understanding of the dynamic changes in macrophages during IPF. Systematic research is needed to understand macrophage heterogeneity and their various roles.

Several studies have reported metabolic disturbances in lung tissues under fibrotic conditions (11–13). Hence, metabolism-based therapies are emerging owing in part to the disappointing outcomes of several inflammation-based pulmonary fibrosis therapies (14). Metabolic reprogramming directs macrophage activation in various inflammatory conditions. In fact, metabolic changes in macrophages have also been confirmed in cystic fibrosis (15, 16). These results highlight the necessity of systematic investigations into the dynamic metabolic function of macrophages during pulmonary fibrosis progression.

In this study, we constructed a dynamic single-cell landscape of lung immune cells from four different stages of bleomycin-induced pulmonary fibrosis. By systematic comparison, we revealed the diverse roles of macrophages. Analysis of interacting ligands and receptors revealed a highly dynamic signal connection network and highlighted the distinctive role of CCL5-CCR5/CCR1 signalling in the late fibrotic stage. We then re-clustered and analysed subsets of AM and mo-Mac, revealing several profibrotic populations with differential trajectories. Importantly, we identified the different metabolic functions of macrophages at different stages of pulmonary fibrosis. Finally, we reanalysed a human IPF single-cell dataset, detected numerous similarities with our dataset, and identified a potential therapeutic target for future treatment. Taken together, our data provide novel insights regarding the function of macrophages and their subpopulations at different fibrotic stages, facilitating future treatment exploration.

## 2 Materials and methods

### 2.1 Establishment of fibrosis mouse model and single-cell preparation

Male wild-type C57BL/6 mice were purchased from Tsinghua University and housed in the Tsinghua University animal facility under specific pathogen-free barrier conditions. The animal experiments were approved by the Institutional Animal Care and Use Committee of Tsinghua University (approval number: 19-WL4). Mice aged 8 weeks were administered 1.5 mg/kg of bleomycin (Selleck, China) in 40 µL of saline or saline alone via an intratracheal injection. The mice were euthanised by CO_2_ inhalation; subsequently, cardiac puncture was performed and the mice were perfused with 20 mL of phosphate-buffered saline to remove blood cells. Saline-treated mice served as the control group (day 0), other mice were sacrificed 7, 14, and 21 days after BLM administration, each time point had 3 mice (n=3). The lungs were harvested and digested with collagenase A (0.4 mg/mL) and DNase (0.4 mg/mL) to generate single-cell suspensions. Cells generated from same time point were mixed. All cells were blocked with anti-CD16/32 and labelled by anti-CD45 antibody (Biolegend). CD45^+^ immune cells were then isolated using fluorescence-activated cell sorting (FACS) for downstream analysis.

### 2.2 Crosstalk analysis and statistics

Potential cellular crosstalk was inferred from ligand–receptor expression. The ligand–receptor pair dataset for mice was derived from iTALK (17). The interaction score between the two cell types of each ligand–receptor pair was calculated as previously reported (17). The overall interaction scores were log2-transformed to the overall number of ligand–receptor interactions between each cell type pair and calculated separately for each day. The matrices were visualised as a heat map using the Python matplotlib package. Ligand–receptor interactions were visualised as circus plots and bubble plots using the Python Pycircos package and matplotlib, respectively.

After identifying highly expressed or differentially expressed genes, these were matched and paired using our ligand–receptor database to identify significant interactions. To identify alterations in singling interactions, the expression levels of both ligands and receptors were evaluated.

### 2.3 Metabolic pathway analysis

Metabolic pathways analysis was established following previously reported methodologies (18). To be specific, gene set variation analysis (GSVA) was used to calculate the enrichment scores of each metabolic pathway in each cell. The pathway score of a cell group was defined as the mean of the pathway scores for all cells in the group.

The statistical significance of the metabolism score was calculated as follows:


$$p\left(m,C,d\right)=Wilcoxon(\{m_{c}|c\in C,\;d_{c}=d,\{m_{c}|c\in C,d_{c}\neq d\}),$$

where p() is the *P*-value; m is the metabolic pathway; C is the cell type; d is the sampling time point; c is a single cell; $m_{c}$ is the GSVA score of pathways m of cell c ; and $d_{c}$ is the sampling time point of cell c . *P*-values were adjusted using the false discovery rate (FDR) method, and pathways with FDR ≤ 0.05 were considered significantly higher or lower at a given time point than at other time points.

### 2.4 Trajectory analysis and RNA velocity

Trajectory analysis was performed using the ‘Monocle’ package. Highly variable genes were selected by fitting a curve to the mean gene expression and empirical dispersion (dispersion table). Genes with mean expression >0.1 and empirical dispersion larger than the fitting result were retained. Dimensionality reduction and trajectory reconstruction were then performed (reduceDimension, max_components = 2, method = ‘DDRTree’). The pseudotime of the cells was calculated by setting the branch with the greatest number of D0 cells as the root state and then ordering the cells.

For RNA velocity analysis, we loaded our single-cell RNA sequencing data of all mice macrophages, into an AnnData object which is the data structure used by scvelo. Then, we preprocess our scRNA-seq data using scvelo’s preprocessing functions. This step typically involves quality control, normalization, and dimensionality reduction. After preprocessing the data, we estimated RNA velocity using scvelo’s tl.recover_dynamics function. This function infers the unspliced and spliced mRNA counts for each cell, which are used to compute RNA velocity vectors. Once the dynamics are recovered, we compute RNA velocity using scvelo’s tl.velocity function. Finally, we use velocity_embedding_stream function to generate a plot showing the RNA velocity vectors as arrows overlaid on a UMAP projection of the cells. All steps were processed with default parameters.

### 2.5 Human and mouse data analysis and comparison

Single-cell data sampled from patients with IPF were obtained from a publicly available dataset (10). We selected data sampled from individuals who were labelled as either ‘IPF’ or ‘donor’. The data underwent standard scRNA-seq data processing steps such as cell filtering, normalisation, scaling, principal Components Analysis (PCA), harmony, clustering, and uniform manifold approximation and projection (UMAP) visualisation. Clusters that expressed protein tyrosine phosphatase receptor type C (*PTPRC*) with mean normalised counts >1 were identified as immune cells. Immune cells were then assigned rough cell types by manual annotation using the markers listed in **Supplementary Table 5**. Marker genes of human immune cell types were calculated using the Wilcoxon test. We retained the orthologous gene pairs if the human gene was a significant marker in a human cell type, and if the mouse gene was also a significant marker in the same mouse cell type. The results are presented in **Supplementary Table 6**.

### 2.6 Gene set enrichment analysis

We used GSEA (19) to identify whether genes highly expressed in the IPF sample of human macrophages were also highly expressed in mouse macrophages from the day 21 sample. We first calculated the log2 fold change of genes (D21 versus D0), sorted the genes in descending order, and used these genes as the background gene set. GSEA (20) was then applied to identify whether the highly expressed genes in the macrophages of IPF patients were enriched.

### 2.7 Alignment, quantification, and quality control of scRNA-seq data; data integration, normalisation, clustering, and annotation; detection of doublets and dead cells; and enrichment analysis

Procedures are detailed in **Supplementary Methods**.

## 3 Results

### 3.1 scRNA-Seq profiling defines the immune landscape during pulmonary fibrosis progression

**Figure 1:**
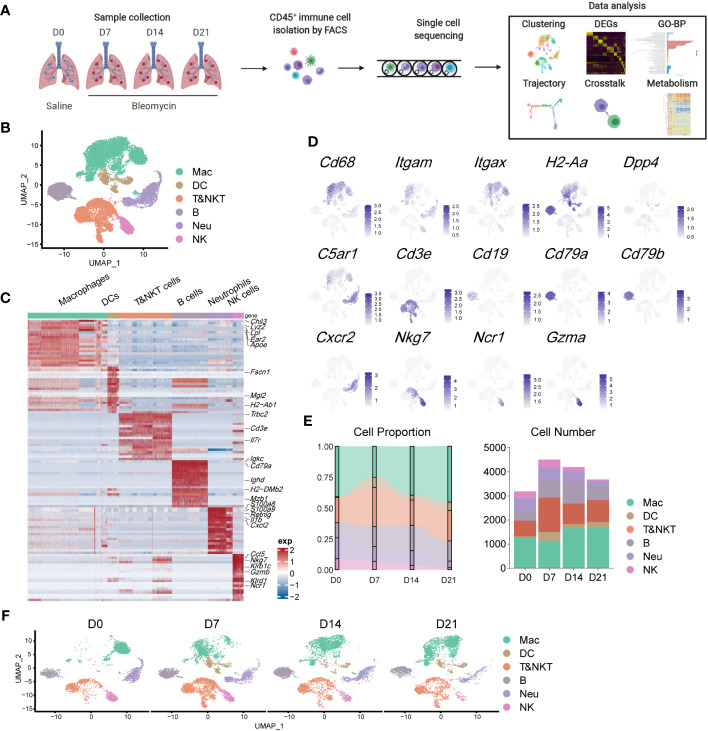
Overview of the immune landscape during the progression of bleomycin-induced pulmonary fibrosis (BIPF). **(A)** Schematic of the experimental design. **(B)** Uniform manifold approximation and projection (UMAP) analysis of immune cells in the IPF lung; coloured by unsupervised clustering results with cell type annotations on the right. **(C)** Heatmap of the top 20 highly expressed genes for each immune cell type; representative marker genes are labelled. **(D)** Canonical cell markers were used to label different cell clusters as represented in the UMAP plot. **(E)** Bar plots deciphering the percent of different types of immune cells in total immune cells at different stages of bleomycin-induced pulmonary fibrosis. **(F)** UMAP plots depicting the projection of cells from various stages of pulmonary fibrosis on a 2D map.

To evaluate the contribution of immune cells to pulmonary fibrosis progression, we utilized a murine model of bleomycin-induced idiopathic pulmonary fibrosis (BIPF) to mimic human IPF (**Figure 1A**; **Supplementary Figure 1**). Lung CD45^+^ immune cells were isolated using fluorescence-activated cell sorting from naïve mice (day 0 [D0]), and at the following stages after bleomycin administration: acute inflammatory phase (D7), profibrotic phase (D14), and later fibrotic phase (D21; **Figure 1A**). In total, we obtained 15,596 cells using 10× Genomics Chromium droplet single-cell RNA sequencing (scRNA-seq; **Figure 1A**). These cells were normalised and clustered; the clusters were then annotated based on their specific gene expression markers: macrophages (CD68^+^C5ar1^+^), dendritic cells (CD11c^+^MHC-II^+^Dpp4^+^), T and natural killer T (NKT) cells (CD3e^+^), B cells (CD19^+^CD79a/b^+^), neutrophils (CXCR2^+^C5ar1^+^), and NK cells (NKg7^+^NCR1^+^Gzma^+^) (**Figures 1B–D**). When compared with the naïve control (D0), the proportion of macrophages in CD45^+^ immune cells initially reduced on day 7, but rapidly recovered by days 14 and 21 (**Figure 1E**). The proportions of DCs, B cells, and T&NKT cells increased, while those of neutrophils and NK cells decreased (**Figure 1E**). Although the frequency of each immune cell did not markedly change during BIPF progression, projection data showed that the composition of each immune cell population, especially macrophages, varied at different stages (**Figure 1F**). These data indicate that immune cells, particularly macrophages, have different subpopulations or different activated statuses at different BIPF stages.

### 3.2 Ligand–receptor analysis reveals the central role of macrophages in interaction networks of immune cells during pulmonary fibrosis progression

**Figure 2:**
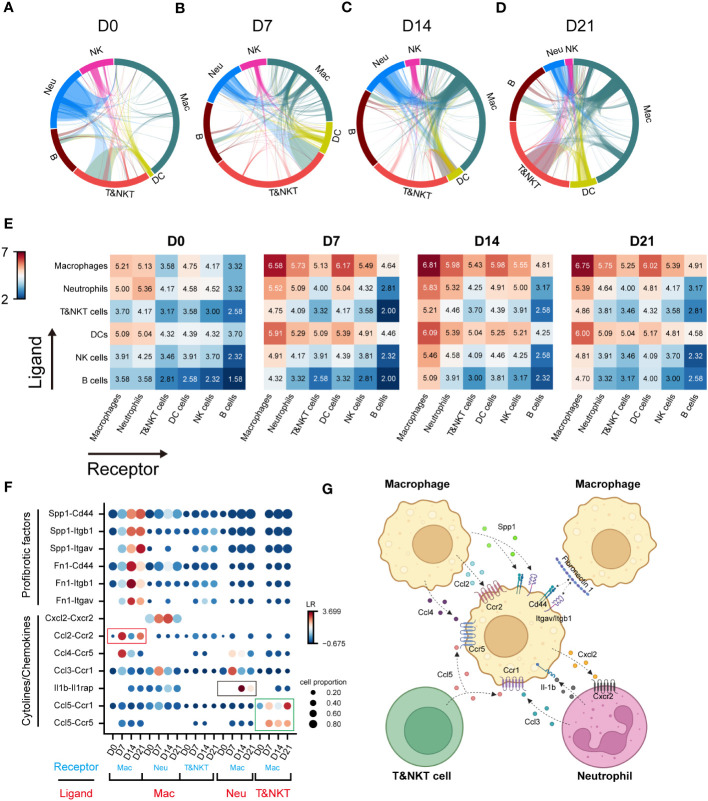
Communication networks between lung immune cells during the progression of pulmonary fibrosis. **(A–D)** Network plots showing the interaction strength among immune cell subtypes. Cell types and interactions are depicted as nodes and edges, respectively. Nodes are coloured by cell identity and sized by the number of interactions. Edge thickness indicates the number of interactions between the connecting cells. Edges are coloured red if they are ranked in the top 15% of all edges in the plot. **(E)** Heatmap showing the interaction score (Log2LR-pairs) among different immune cell subtypes. For each heatmap, rows indicate transmitters, and columns indicate responders. Red indicates a high interaction score. **(F)** Expression of cell-type-specific ligand-receptor interactions inferred by iTALK. Shown are predicted interactions between macrophages, neutrophils and T&NKT cells at different stages. Circle size indicates the proportion of ligand-receptor expression in interacting cells and circle colour indicates mean expression of ligand and receptor genes for interacting pairs. **(G)** Predicted regulatory network between macrophages, neutrophils and T&NKT cells. Created with BioRender.com.

Cell-cell interactions constitute a significant component of cellular function, mediated by ligand-receptor interactions (21). The progression of IPF is intricately linked to the functioning of immune cell, with interactions among these immune cells constituting a crucial component. However, these interactions have not been extensively explored to date. To elucidate the role of macrophages in communication networks during BIPF progression, we used a well-established pipeline, iTALK, to identify potential cell–cell interactions (17). The Network circle plots showed key interaction through different cell types. The interaction link start from a ligand and end in a receptor, and the thickness represents the interaction strength. Myeloid immune cells, including macrophages, dendritic cells, and neutrophils, were the major transmitters noted to have strong interactions with each other in the steady state (D0) (**Figure 2A**). However, with pulmonary fibrosis progression, macrophages exhibited a significantly increased strength of interactions with other immune cells (**Figures 2B–D**). Conversely, few interactions were found involving T&NKT, B and NK cells (**Figures 2A–D**). We then calculated the communication scores in each cell pair during fibrosis progression. Moreover, macrophages derived more ligands in inflammatory and fibrotic lungs, where they interacted with receptors expressed on T and NKT cells, neutrophils, and other macrophages (**Figure 2E**). By contrast, the predicted interactions between neutrophils and other immune cells were the strongest during the acute inflammatory phase, but declined later (**Figures 2A–E**). Meanwhile, the strength of the interaction between dendritic cells and other immune cells was relatively lower than that of macrophages (**Figures 2A–E**).

We further predicted the functional interactions between macrophages and other immune cells, and classified them as macrophages as ligand and macrophages as receptor. The result showed that macrophages expressed high levels of profibrotic factors, including FN1 and SPP1 during fibrosis. These factors bound to ITGAV/ITGB1 and CD44 within macrophages (**Figure 2F**), which was consist with previous reports (22, 23). Significant changes were also detected in other interactions involving cytokines and chemokines, such as CCL2, CCL4, and CXCL2 (all expressed by macrophages), CCL5 (expressed by T and NKT cells), CCL3, and IL-1β (expressed by neutrophils; **Figures 2F, G**). Notably, a large proportion of interactions involving cytokines and chemokines were highly active in the inflammatory phase (D7), and declined in the later phase (D14–D21), such as CCL2-CCR2 (Mac-Mac, red frame, **Figure 2F**). We also observed similar pattern for IL1β-IL1RAP interactions (Neu-Mac, black frame), which reached peak at D14 (**Figure 2F**). However, CCL5-CCR5/CCR1 (T&NKT-Mac, green frame) signalling was continuously active, which might suggest that this signal have a distinctive role in late fibrotic stages of IPF, consistent with the observed late activation of CCL5 (24, 25). Taken together, these data reveal the major regulatory role of macrophages in immune cells and predict that CCL5-CCR5/CCR1 may serve as a potential therapeutic target for lung fibrosis (**Figures 2G**).

### 3.3 Dynamic changes occur in tissue-resident and infiltrating macrophage clusters during pulmonary fibrosis progression

**Figure 3:**
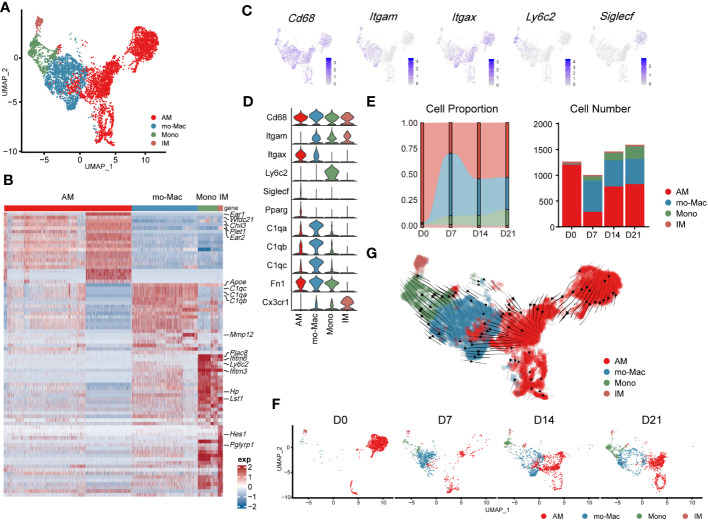
Transcriptomic characteristics of macrophages from different cell origins in the lung during the progression of pulmonary fibrosis. **(A)** UMAP plot depicting the subcluster of macrophages in the lung of bleomycin-induced pulmonary fibrosis. **(B)** Heatmap of the top 20 highly expressed genes for each subcluster of macrophages; representative markers genes are labelled. **(C)** UMAP plot showing the specific marker gene expression in the subcluster of macrophages. **(D)** Violin plots comparing the expression level of selected genes in each cluster of mouse macrophages. **(E)** Bar plots depicting the composition of different subclusters of macrophages at various stages of bleomycin-induced pulmonary fibrosis. **(F)** UMAP plots depicting the projection of macrophages from various stages of pulmonary fibrosis on a 2D map. **(G)** Trajectory of differentiation within all macrophages f predicted by RNA velocity.

Further compartmental analysis using higher-resolution clustering identified four subclusters (**Figure 3A**), mainly according to the expression of *Itgam*, *Itgax*, *Ly6c2*, and *Siglecf*. They were annotated as follows: IMs (Itgam^+^Itgax^-^), monocyte-derived macrophages (mo-Macs; Itgam^high^Itgax^+^Siglecf^low^C1qa,b,c^high^), Ams (Pparg^+^Itgam^low^Itgax^+^Siglecf^mid/high^), and monocytes (Itgam^high^Itgax^-^Ly6c2^high^) (**Figures 3A–D**). Ams and mo-Macs were the two major populations; their proportions and that of their subclusters, changed according to pulmonary fibrosis progression (**Figures 3E, F**). AMs comprise a special group of macrophages that have relatively unique gene expression profiles, functions and origin (26). Mo-Macs originate from infiltrating monocytes, both of which migrated into the lungs during inflammatory responses (**Figures 3E, F**), as previous reported (27). To further understand the differentiation trajectory of macrophage subsets, we applied RNA-velocity analysis on total macrophages to predict the path. Our results suggested that after fibrosis onset, monocytes could differentiate into mo-Mac, while AMs had an internal transition trajectory and IMs showed no obvious differentiation trajectory from other cells (**Figure 3G**). This result suggested that mo-Mac exhibited migration characteristics, while AM clusters had the potential to transform within AMs.

We also analysed the gene expression profiles of AMs, mo-Macs, and IMs at different stages. In the steady state (D0), AMs expressed high levels of signature genes, such as *Fabp1*, *Ear1*, and *Krt19.* However, in the acute inflammatory stage they exhibited a different gene expression profile, with upregulated expression of profibrotic genes, including *Fn1*, *Spp1*, and *Cd63* (D7). Notably, expression of these genes remained high in the later stages (D14–D21; **Supplementary Figure 2A**). mo-Macs infiltrated the lungs from D7 and expressed high levels of *Ccl4, Cxcl10*, and *Nfkbia*, reflecting an activated inflammatory phenotype (28) (**Figure 3E**; **Supplementary Figure 2A**). mo-Macs potentially manifested a profibrotic phenotype, substantiated by the high expression of profibrotic genes, notably including *Mmp12*, *Lpl*, *Mmp19*, and *Ecm1* (**Supplementary Figure 2A**).

Moreover, the scRNA-seq data showed that IMs existed in the patient’s lungs at all time points; their populations were small and rarely changed during pulmonary fibrosis progression (**Figure 3E**). In a healthy state, IMs express high levels of *Ptger4* and *Ifi27l2a* and are involved in the regulation of the innate immune response (29). In the acute inflammatory stage, their ability to recruit monocytes is enhanced by the expression of *Ccl2* (30). Like AMs, *Spp1* and *Apoe* were also expressed by IMs on D14 and D21 (**Supplementary Figure 2A**). This finding shows that IMs may have the potential to promote IPF at the level of gene expression, aligning with contemporary perspectives that IMs are associated with IPF progression (31, 32)

### 3.4 Analysis of alveolar macrophage identifies specific clusters associated with disease state and their developmental trajectories

**Figure 4:**
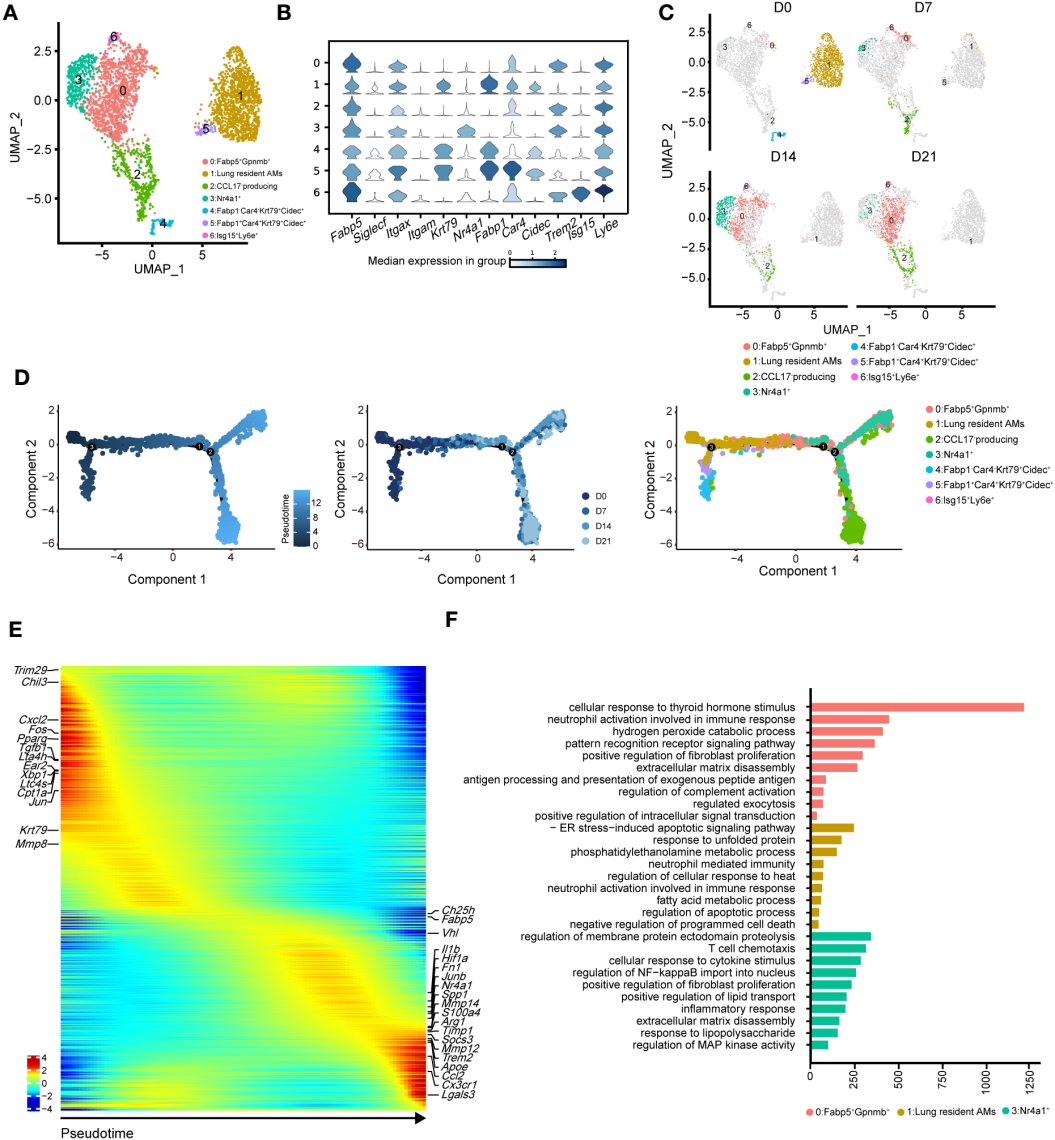
Heterogeneity of alveolar macrophages (AMs) during the progression of pulmonary fibrosis. **(A)** UMAP plots depicting the subcluster of AMs in the lungs with bleomycin-induced pulmonary fibrosis. **(B)** Violin plots comparing the expression levels of selected genes in each cluster of alveolar macrophages. **(C)** UMAP plots depicting the projection of AMs from different stages of pulmonary fibrosis on a 2D map. **(D)** DDRTree plot showing the trajectory development of AM; the plots are coloured by derived pseudotime (left), Seurat clustering result (middle), and sampling timepoint (right). **(E)** Gene expression profiles of AMs ordered according to the pseudotime trajectory, with the x-axis representing pseudotime. Fibrosis-related genes are labelled. **(F)** Functional enrichment analysis of GO biological processes, with significant marker genes of clusters 0, 1, and 3 of AMs.

To precisely identify the primary functional population of AMs during pulmonary fibrosis progression, we further divided AMs into seven clusters (**Figures 4A, B**). The projection data showed that clusters 1, 4, and 5 were lung-resident cells only found at steady state (D0, **Figure 4C**). These cells expressed high levels of AM-specific markers, including *Fabp1*, *Cidec*, and *Krt79* (**Figure 4C**, **Supplementary Figure 3A**). After D7, AMs were replenished by clusters 0, 2, and 3 (**Figure 4C**; **Supplementary Figure 3B**). These clusters shared similar profibrotic gene expression profiles, including *Spp1* and *Fn1* (**Supplementary Figure 3A**). Notably, cluster 0, characterised by high *Gpnmb* expression, was continuously augmented as the primary AM population in fibrotic lungs (D21). Meanwhile, cluster 2 expressed proliferation genes, including *Pclaf*, *Stmn1*, and *Top2a* (33), and cluster 3 harboured a proinflammatory phenotype with *Jun* and *S100a4* expression (**Supplementary Figure 3A**). Since the populations of cluster 2 and 3 peaked on D7 and D14, respectively, our data indicate different populations involved in maintaining inflammatory and fibrotic phenotypes. Furthermore, we identified a rare population in cluster 6, which expressed *Ifi211*, *Isg15*, and *Trim30a*; these genes are associated with defence against viral infection (34); however, their role in pulmonary fibrosis is unknown (**Figure 4B**; **Supplementary Figure 3A**).

We next performed pseudotime inference of AMs during pulmonary fibrosis progression (**Figure 4D**). Cells were clustered according to different pseudotime, sampling timepoints and clusters. The trajectory path of AMs started with lung-resident AMs (clusters 1, 4, and 5 on D0), and then bifurcated into proinflammatory and profibrotic AMs either in the profibrotic phase (clusters 0, 2, and 3 on D14) or in the later fibrotic phase (clusters 0 and 2 on D21; **Figure 4D**, middle and right). Our result might suggest that AMs population at homeostasis might exhibit self-renewal and differentiation properties. We also investigated changes in AM gene expression profiles and found that certain metabolism- and proliferation-related genes, such as *Cpt1a* and *Krt79* (35), were highly expressed at the early pseudotime, while proinflammatory and profibrotic genes, such as *Fn1* and *Spp1*, were upregulated later (**Figure 4E**). We also found that *Trem2* was upregulated in AMs; however, its function in IPF remains unknown. These results further reflect the necessity of distinguishing AM subgroups and revealing their associations with fibrotic progression.

Using Gene Ontology (GO), we performed functional enrichment analysis on the major clusters 0, 1, and 3. Consistent with the gene expression profiles, the fatty acid metabolism pathway was enriched in cluster 1 (**Figure 4F**). By contrast, biological processes associated with fibrosis, including ‘positive regulation of fibroblast proliferation’ and ‘extracellular matrix disassembly’, were enriched in cluster 0 (**Figure 4F**). Cluster 3 harboured the functional pathways for regulating the immune response and promoting fibrosis. We also compared the expression of key functional genes and found that cluster 3 was expected to secrete more *Cxcl2*, *Cxcl1*, and *Il1b* than the other clusters (**Supplementary Figure 3C**). Together, these data described the dynamic changes in AM subclusters and identified cluster 0 with *Gpnmb* expression as a potential target in pulmonary fibrosis treatment.

### 3.5 Diverse clusters of monocyte-derived macrophages shows distinct functional features during pulmonary fibrosis progression

**Figure 5:**
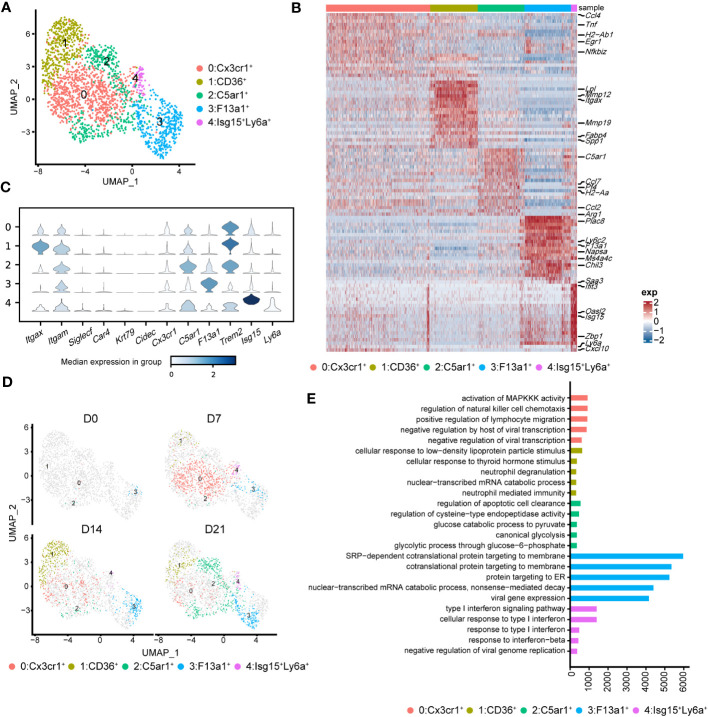
Heterogeneity of monocyte-derived macrophages (mo-Mac) during the progression of pulmonary fibrosis. **(A)** UMAP plots depicting the subcluster of mo-Mac in lung of bleomycin-induced pulmonary fibrosis. **(B)** Heatmap of the top 20 highly expressed genes of each cluster of mo-Mac; representative markers genes are labelled. **(C)** Violin plots comparing the expression level of selected genes in each cluster of alveolar macrophages. **(D)** UMAP plots deciphering the projection of mo-Mac from different stages of pulmonary fibrosis on a 2D map. **(E)** Functional enrichment analysis of GO biological processes with the significant marker genes of each cluster of mo-Mac.

To better understand the pathological roles of mo-Mac, we identified five clusters using higher-resolution clustering (**Figures 5A, B**). Cluster 0, marked by high levels of *Cx3cr1*, was the primary population in the acute inflammatory phase (D7), expressing inflammation-related genes, such as *Ccl4* and *Tnf* (**Figures 5B, C**). During the profibrotic phase (D14), cluster 0 was replaced by cluster 1, termed CD11c^+^CD11b^+^CD36^high^ mo-Macs, which expressed higher levels of profibrotic genes, including *Lpl*, *Mmp12*, *Mmp19*, and *Spp1* (**Figures 5B–D**). Cluster 2, termed C5ar1^high^ mo-Macs, was a dominant population on D21 with a uniquely high expression of *Ccl7*, *Pf4*, *Ccl2*, and *Arg1* and exhibited a strong ability to regulate leukocyte migration (**Figures 5B–D**). Meanwhile, the proportion of cluster 3 increased along with BIPF progression, expressing both monocyte markers (*Ly6c2*), and macrophage markers (*F13a1*; **Figures 5B–D**). We also identified a rare population in cluster 4, which expressed higher levels of *Isg15*, related to anti-virus responses (**Figures 5B–D**).

We also performed pseudotime inference on mo-Macs. Unlike in AMs, cells from different timepoints could be found at the beginning and end of the trajectory, indicating a lack of obvious correlation among mo-Mac clusters (**Supplementary Figure 4B**). We also analysed the gene expression profiles and found that proinflammatory genes, such as *Ccr2* and Il1b, tended to be expressed in the early phase, while profibrotic genes, such as *Spp1* and *Arg1*, tended to be expressed in the later phase (**Supplementary Figure 4C**). Functional enrichment analysis of each cluster revealed more diverse roles for mo-Macs; cluster 0 had high levels of *Ccl4* and was associated with immune responses, including the regulation of lymphocyte migration (**Figure 5E**; **Supplementary Figure 3C**). Meanwhile, cluster 1 expressed higher levels of *Il1rn* and *Lgals3*, participating in ‘neutrophil mediated immunity’ (36) and leading to tissue inflammation (**Figure 5E**; **Supplementary Figure 3C**). Cluster 2 was significantly correlated with glucose metabolism (**Figure 5E**). Taken together, these data identified CX3CR1^+^ mo-Macs (cluster 0) as the major proinflammatory population in the early phase of fibrosis (D7), whereas CD36^+^ mo-Macs (cluster 1) were the major profibrotic populations in later phases of fibrosis (D14, D21).

### 3.6 Metabolic features of macrophages change with pulmonary fibrosis progression

**Figure 6:**
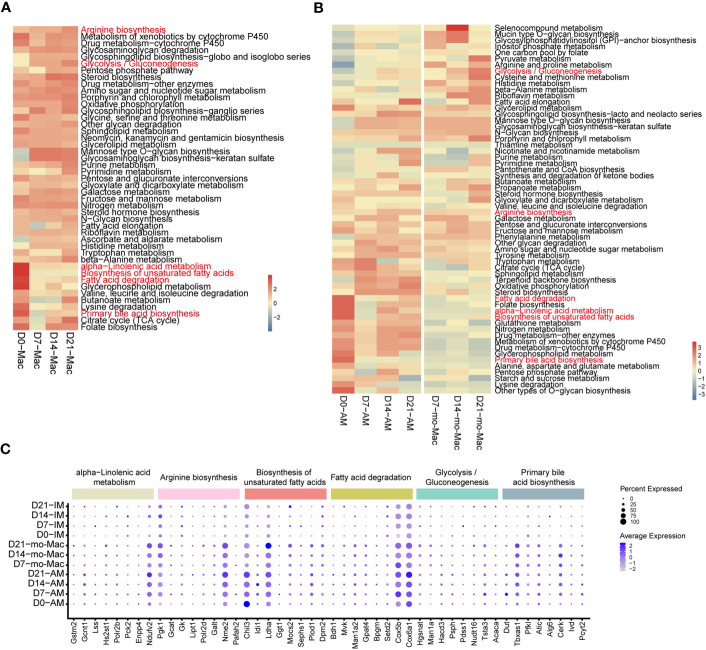
Single-cell RNA-seq analysis reveals specific metabolic signatures of macrophages in the progression of pulmonary fibrosis. **(A)** Heatmap showing the selected metabolic pathway activity of immune cell types at different stages of pulmonary fibrosis. Red indicates strong metabolic activity. **(B)** Heatmap showing the selected metabolic activity of the different subclusters of macrophages at different stages of pulmonary fibrosis. **(C)** Dot plot depicting the percentage of cells expressing the gene and the expression levels of genes in the cells expressing these genes. Genes with most variation level related to specific metabolic pathways were selected.

Cellular metabolism shapes macrophage phenotype and function (37), we would like to know if the metabolic features of immune cells, especially macrophages, were changed. To investigate the metabolic changes in immune cells during pulmonary fibrosis progression, we analysed the enrichment of metabolic pathways in different immune cells according to a published computational pipeline (18). Most of the 80 metabolic pathways were highly enriched and dynamic in macrophages compared to that in other immune cells (**Supplementary Figure 5A**). We noticed that biosynthesis of unsaturated fatty acid, fatty acid degradation, alpha-linolenic acid metabolism, and primary bile acid biosynthesis sharply decreased in macrophages during pulmonary fibrosis progression (**Figure 6A**). In addition, the citric acid cycle (TCA cycle) and glycolysis/gluconeogenesis process in macrophages changed with disease progression, reaching peak activity in the fibrotic phase (D21; **Figure 6A**). This likely indicates that during fibrosis, the metabolic status of macrophages transforms from lipid metabolism to glucose metabolism, consistent with the results of former study stating that glucose consumption is upregulated in an IPF mouse model (38). In addition, we noticed that arginine synthesis and metabolism were enhanced, which has been shown to correlate with lung fibrosis (39). Further quantification of the changes in metabolic pathways in macrophage subsets found that AMs and mo-Macs showed higher metabolic activity and diversity than did monocytes and IMs (**Supplementary Figure 5B**). Specifically, when fibrosis occurred, lipid-related-metabolism pathways firstly decreased and were then slightly recovered in AMs, such as fatty acid degradation and biosynthesis of unsaturated fatty acids. While some glucose-related-metabolism pathways increased in mo-Macs, especially for glycolysis/gluconeogenesis but not Oxidative phosphorylation. The activities of other types of metabolism pathway, like alpha-linolenic acid metabolism and primary bile acid biosynthesis were all lower under disease condition, which were primarily found in AMs (**Figure 6B**).

Since we quantified the metabolic feature by evaluating a series of gene expression profiles, we would like to investigate which gene changes are causing such results. We therefore analysed gene expression changes associated with the metabolic pathways mentioned above by selecting genes with the greatest variation in current metabolic pathway. Consistent with changes in metabolism, we observed decreased expression of genes associated with certain metabolic pathways, such as fatty acid degradation, and increased expression in other pathways, such as glycolysis/gluconeogenesis (**Supplementary Figure 5C**). Further characterisation of gene expression in macrophage subclusters revealed the different regulatory function of genes on distinct cells. To be specific, the expression of *Chil3* related to the biosynthesis of unsaturated fatty acids decreased at D7 in AMs, but gradually increased from D7-D21 in mo-Macs, indicating a strong regulatory ability of *Chil3* in macrophage metabolism (**Figure 6C**). All gene expression changes within clusters of interest are presented in **Supplementary Figure 5**.

### 3.7 Macrophage subsets with similar gene expression features occur during pulmonary fibrosis in mice and humans

To further evaluate our findings, we reanalysed published scRNA-seq data from patients with IPF (10). As expected, we identified several known major immune cell lineages: myeloid cells (neutrophils, DCs, and macrophages) and lymphocytes (T and NKT cells, NK cells, and B cells) (**Supplementary Figure 6A**). These human immune cell subsets exhibited strong similarities in gene expression profiles to those observed in mice (**Supplementary Figure 6B**).

**Figure 7:**
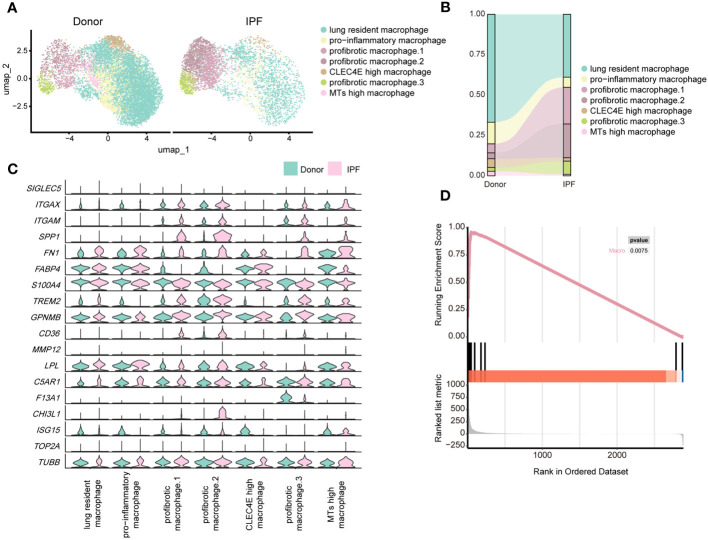
Single-cell RNA-seq analysis reveals similar profibrotic macrophage subclusters between the lungs of bleomycin-induced pulmonary fibrosis mouse models and those of patients with IPF. **(A)** UMAP plot depicting the subclusters of macrophages in patients with IPF and healthy donors. **(B)** Distribution of immune cells in normal and fibrotic lungs of mouse and humans. **(C)** Violin plots comparing the expression level of selected genes in each cluster of human macrophages. **(D)** Similarity score for comparing macrophages in the fibrotic mouse and patients with IPF.

By re-clustering human macrophages, we identified seven clusters based on their gene expression profiles (**Supplementary Figure 6C**; **Figure 7A**). Most lung-resident macrophages, proinflammatory macrophages, CLEC4E-high macrophages, and MT-high macrophages were from lungs of non-fibrosis donors (**Figure 7B**). These clusters were similar to those of mice on D0 and are likely associated with maintenance of lung homeostasis. Among them, proinflammatory macrophages expressed higher levels of chemokines, indicating an ability to recruit inflammatory cells (40). CLEC4E-high macrophages harboured high expression of *CLEC4E*, *BCL2A1*, and *MDM2* (**Supplementary Figure 6D**), which are related to the cellular inflammatory response and inhibition of apoptosis (41). Interestingly, a new cluster expressing high levels of MT-related genes, termed MT-high macrophages, was primarily observed in the lungs of donors and may have anti-apoptotic effects (42); however, their roles in pulmonary fibrosis remain unclear (**Supplementary Figure 6D**).

Three subpopulations of macrophages were significantly increased in fibrotic lungs and shared similar profibrotic gene signatures, including *SPP1*, *TIMP1*, *FN1*, and *CCL2* (**Figure 7C**; **Supplementary Figure 6D**), which resemble macrophages from the profibrotic and fibrotic phases of our mouse model (D14 and D21). Profibrotic mac.1 expressed high *CCL2*, indicating a strong ability to recruit monocytes/macrophages and mediate inflammatory response during IPF progression (**Supplementary Figure 6D**). Profibrotic mac.2 exhibited higher *MMP9* and *MMP7* expression (**Supplementary Figure 6D**), and had specific expression of *CHI3L1*, which are thought to play crucial roles in tissue remodelling (43, 44). In addition, both profibrotic mac.1 and 2 expressed high levels of *TGFB1*, further suggesting that they might have the characteristics of typical M2 macrophages. And profibrotic mac.2 were more similar to M2c macrophages given their expression of *MMP* genes (45). Similar to cluster 3 comprising mo-Macs in our mouse study, profibrotic mac.3 expressed high levels of *F13A1*, as well as *MS4A6A*, which are expressed by M2 macrophages with a highly active inflammatory response (46) (**Figure 7C**; **Supplementary Figure 6D**). Furthermore, we observed upregulated expression of *GPNMB* in all macrophage subsets and *CHI3L1* in profibrotic mac.2, indicating their significant roles in IPF progression. We further analysed the upregulated genes in mouse macrophages and compared them with those in human IPF macrophages. We collected the up-regulated genes in mouse lung macrophages (Gene set A, D21 versus D0), and compared those with the up-regulated genes in human lung macrophages (Gene set B, IPF versus donor) by applying the GSEA. The result reflected that gene set B was enriched in Gene set A (*P*< 0.01), indicating the expression profiles change of mouse lung macrophages was similar to the expression profiles change of human macrophages (**Figure 7D**; **Supplementary Table 7**).

Moreover, in line with the metabolic features of macrophages in the mouse model, the metabolic activities of macrophages in human lungs were more vigorous compared with other immune cells. And more metabolic pathways were found to be up-regulated in macrophages of IPF patients, compared to other immune cells. (**Supplementary Figure 7A**). High consistency was also observed in the changes of the metabolic status of macrophages between humans and mice, as evidenced by similarly enhanced TCA cycle and glycolysis/gluconeogenesis activity and decreased alpha-linolenic acid metabolism (**Supplementary Figure 7A**). Analysis of macrophage subpopulations suggested these changes were attributed to profibrotic mac.2. (**Supplementary Figure 7B**). This finding suggests that similar metabolic alterations in lung macrophage subpopulations also exist in IPF patients, although this remains to be confirmed experimentally.

## 4 Discussion

IPF is a typically progressive interstitial lung disease with limited therapeutic strategies (47). Recently, new insights into the role of macrophages have highlighted their potential as anti-inflammatory therapeutic targets for IPF (48). However, it is crucial to understand how the immune microenvironment, especially macrophages, in the lungs changes with disease progression and to define optimal intervention timing and targets. In this study, we applied single-cell RNA-seq analysis to a BIPF mouse model to portray the immune cell landscape in the lungs at different stages of pulmonary fibrosis progression. Our analysis revealed distinct components of immune cells associated with different stages of pulmonary fibrosis progression. In particular, macrophages were the primary immune cells that exhibited close communication with other immune cells during disease progression. Moreover, *Gpnmb* and *Trem2* upregulation in macrophages was found to potentially have important roles in the progression of pulmonary fibrosis. Meanwhile, changes in specific metabolic pathways of AMs and mo-Macs were also identified with disease progression. In line with the published data related to human IPF, macrophages in our mouse model shared similar gene expression features and metabolic features with the macrophages in lungs of patients with IPF. Hence, our scRNA-seq analysis of immune cells from the lungs during the different phases of pulmonary fibrosis provides a comprehensive dataset for better understanding the functions of immune cells in the disease process (http://dynamic-ipf.hupo.org.cn/). Indeed, the data generated in this study can be broadly adapted for biomedical and clinical research.

Macrophages are the primary profibrotic immune cells associated with pulmonary fibrosis (4). Moreover, they have been shown to direct the migration and function of other immune cells in different disease settings by producing various cytokines or chemokines (6). Ligand–receptor pair enrichment is an efficient means to analyse the interactions between immune cells during pulmonary fibrosis progression (49, 50). In this study, we showed that the frequencies of macrophage-related interactions were consistently highest and most dynamic during IPF progression, highlighting macrophages as the main component in the immune microenvironment. Further analysis of ligand–receptor pairs revealed that the kernel interaction types were FN1- and SPP1-linked profibrotic adhesion. Recently, the SPP1 and CD44 axes have been characterised for fibroblasts (51). Our results not only suggest this interaction in macrophages of fibrotic lungs but also extend to include FN1 and ITGAV/ITGB1. Our analysis also showed that T cells regulate macrophages through the CCL5-CCR5/CCR1 axis; although this axis is thought to play a significant role, it has not been thoroughly investigated (25). In summary, our results provide a more complete view of the interaction network in the IPF immune microenvironment, and suggest that blocking the interaction between macrophages and other cells may be a novel strategy for IPF treatment.

By examining macrophage subsets, we found that AM and mo-Mac made up the largest proportions and exhibited the greatest level of change during IPF. Further re-clustering results revealed changes other than expression profiles in these subpopulations. AMs were dominant in the healthy state, residing primarily in the lung and expressing metabolism-related genes such as *Ear1* and *Fabp1*. After bleomycin infusion, the steady state AMs quickly disappeared and were replaced by proinflammatory and profibrotic AMs. Specifically, we identified an AM population with potential profibrotic function and *Fabp5* and *Gpnmb* expression; targeting this population may help to provide therapeutic effect for pulmonary fibrosis. It is worth noting that, the AMs included two distinct subsets showed in **Figure 3B**. Through further expression profile analysis at different stage showed in **Supplementary Figure 2A**, we discovered that these two groups correspond to AM clusters 1,4,5 and 0,2,3, respectively. Conversely, mo-Macs were rarely detected in a healthy state and are more related to inflammation. They were observed to infiltrate the lung from D7 and express proinflammatory genes, while later expressing profibrotic genes. Although our results indicate that there are sporadic mo-Macs at D0, mo-Macs is generally considered non-existent at this time, which may be due to the inherent defects during sample preparation, and was left unexplored in the following analysis. Our gene expression profile analysis also revealed IM heterogeneity in healthy and diseased states, consistent with previous research that IM might have a distinct role in both healthy and diseased states, although their population is small (52).

By applying pseudotime analysis, our results suggest a differentiation trajectory in which the lung-resident AMs had the potential to differentiate into proinflammatory and profibrotic AM populations. Some studies indicate that monocytes are the source of AMs in inflammatory conditions (53, 54), and termed those AMs as mo-AMs (monocytes-derived AMs) to distinguish them from TR-AMs (tissue-resident AMs). Their data also showed that TR-AMs composed of large number of total AMs at late fibrosis, which might come from TR-AMs at steady state and consistent with our results. Our data supported that TR-AMs have self-renewal and differentiation properties during fibrosis progression, and these two ideas are not disparate and serve to support each other. This result provides further understanding of the source of proinflammatory and pro-fibrotic AMs during pulmonary fibrosis, which is crucial in exploring the mechanism of IPF. By contrast, we observed no clear internal differentiation pathway among mo-Mac clusters as they were primarily derived from circulating monocytes.

Assessing metabolic changes has attracted considerable research attention owing to their role in the pathogenesis of fibrosis in many organs (14, 55). Cellular metabolism is believed to play a vital role in shaping macrophage phenotype and function (37). Our study provides the first comprehensive description of the metabolic profile of different immune cells during pulmonary fibrosis. In general, more metabolic pathways were enhanced in macrophages of IPF patients, compared to other immune cells, further demonstrating the relationship between macrophage metabolism and pulmonary fibrosis progression. In particular, our results suggest a shift from lipid-related-metabolism to glucose-related-metabolism in macrophages during fibrosis. Since fatty acid metabolism is considered to be closely related to fibrosis (56), our findings support the perspective (57) that metabolic reprogramming, especially in macrophages, represents a potential novel therapeutic approach for IPF. We further confirmed that metabolic changes in macrophages were primarily attributed to AMs and mo-Macs and predicted *Chil3* as a potential key regulatory gene. Meanwhile, certain genes, such as *Cox5b* and *Cox6a1* appeared in different pathways, potentially because they are involved in the mitochondrial respiratory chain and participate in different metabolic pathways.

To relate our findings more closely to the treatment of human IPF, we analysed a published database to compare any similarities between macrophage populations in humans and mice (10). The clustering results differed slightly from previous articles, which may be because we refined the clustering methods to further eliminate the batch effect across different samples. We identified three profibrotic macrophage clusters that expressed similar profibrotic related gene signatures, including *SPP1*, *FN1*, *CHI3L1*, *MMP9*, *MMP7*, and *CCL2*. High expression of *CD14* in these populations supports the theory that they originated from monocytes during IPF progression (58). Many similarities were observed between the clustering results for macrophages in humans and mice. For instance, we identified profibrotic mac.2 with high *CHI3L1* expression in IPF patients, similar to that seen in mo-Macs in mice at D14 and D21. We also identified the *F13a1*-high cluster in mouse and human macrophages, as well as similarities among upregulated genes, such as *TREM2* and *GPNMB*. In recent years, these two genes have received considerable research attention for their activity in tumours and Alzheimer’s disease. Our results suggest that they may also have a significant role in lung inflammation and fibrosis. In addition, we identified activity changes in alpha-linolenic acid metabolism and primary bile acid biosynthesis in human macrophages, which were also found in mice macrophages during fibrosis.

The past single-cell sequencing analysis for pulmonary fibrosis mainly focused on the changes of parenchymal cell function (59–61), while we focused more on immune cells, especially the subclusters of macrophages. We classified the macrophage subpopulation in a more detailed and scientific manner, provided a more comprehensive and in-depth transcriptome map, and suggested some regulatory genes that may be related to the fibrosis progression. However, there were certain limitations noted in this study. Due to the lack of IPF survival data, we were not able to further evaluate the mentioned genes and metabolic pathways for application in preclinical study use. Hence, studies using mouse models are required to determine their therapeutic potential on IPF.

In summary, this study used single-cell RNA-seq analysis to reveal comprehensive information on the dynamic changes of immune cells, particularly macrophages, during the progression of pulmonary fibrosis. To the best of our knowledge, this is the first study presenting such findings. Similarities in the changes of macrophages in human IPF were also observed. Collectively, this study provides new insights into the pathogenic mechanisms of IPF, which are crucial for identifying potential therapeutic targets and designing novel treatments for IPF.

## Data availability statement

The datasets for this study are publicly accessible at https://ngdc.cncb.ac.cn/gsa with the identifier CRA011039. The code necessary to perform analysis and generate figures can be obtained from https://github.com/pluto-the-lost/IPF_immune_progression.

## Ethics statement

The animal study was approved by The Institutional Animal Care and Use Committee of Tsinghua University. The study was conducted in accordance with the local legislation and institutional requirements.

## Author contributions

JL, HG, JM, JCL conducted experiments, analysed data, and wrote the manuscript. YT conducted experiments. CY, ML, YZ, ZL analysed data. LW, JZ, YPZ, XZ conceptualised the project, designed the research, supervised experiments and data analysis, and wrote the manuscript. All authors contributed to the article and approved the submitted version.
