# Supplementary material for: Dynamic atlas of immune cells reveals multiple functional features of macrophages associated with progression of pulmonary fibrosis
Source: Front Immunol. 2023 Sep 13;14:1230266. doi: 10.3389/fimmu.2023.1230266 (PMC10525351; doi:10.3389/fimmu.2023.1230266)
Supplement: Supplementary file 1 [file DataSheet_1.pdf]

Supplementary Material

Dynamic atlas of immune cells reveals multiple functional features of macrophages associated with progression of pulmonary fibrosis

Jiaoyan Lv[1†], Haoxiang Gao[3†], Jie Ma[4†], Jiachen Liu[1†], Yujie Tian[1], Chunyuan Yang[4], Mansheng Li[4], Yue Zhao[6], Zhimin Li[6], Xuegong Zhang[3,5]*, Yunping Zhu[4]*, Jianhong Zhang[1,2]*, Li Wu[1,2]*

[1]Institute for Immunology, Tsinghua-Peking Joint Center for Life Sciences, School of Medicine, Tsinghua University, Beijing 100084, China

[2]Beijing Key Laboratory for Immunological Research on Chronic Diseases, Beijing 100084, China

[3]Department of Automation, MOE Key Laboratory of Bioinformatics; Bioinformatics Division and Center for Synthetic & Systems Biology, BNRist, Tsinghua University, Beijing 100084, China

[4]State Key Laboratory of Proteomics, Beijing Proteome Research Center, National Center for Protein Sciences, Beijing Institute of Life Omics, Beijing 102206, China

[5]School of Life Sciences, Tsinghua University, Beijing 100084, China

[6]Annoroad Gene Technology (Beijing) Co., Ltd., Beijing 100176, China

[†]These authors contributed equally to this work.

*** Correspondence:**
Li Wu
wuli@tsinghua.edu.cn

Jianhong Zhang
zhangjianhong@tsinghua.edu.cn

Yunping Zhu
zhuyunping@ncpsb.org.cn

Xuegong Zhang
zhangxg@tsinghua.edu.cn

1 Supplementary Method

Alignment, quantification, and quality control of scRNA-seq data. The 10x-generated read data were aligned to the mm10 mouse reference genome and quantified to read counts using the Cell Ranger Single-Cell Software Suite (V3.0.1, 10x Genomics). Barcodes with expressed gene numbers less than 800 were removed. Genes that were expressed in fewer than 30 cells were excluded. This step generated a cell number of ~17,193, with a median gene number of 1,310.

Data integration, normalization, clustering, and annotation. Downstream analysis was based mainly on the Seurat package. Data from four time points was integrated using Integrate Data, with "RNA" assay set as the default assay. Integrated cells underwent data normalization (Normalize Data, default parameters), data scaling (ScaleData, for all genes), variable gene identification (FindVariableFeatures, vst method), PCA (RunPCA, on the top 1000 genes), and graph-based clustering (FindClusters, on the first 25 PCs, resolution = 0.25). Markers of each cluster were identified using FindAllMarkers (Wilcoxon test, fold change threshold = 0.1). Manual annotation was performed using the markers listed in supplementary table 1. After coarse annotation, cells were classified into general cell types. The cells from each cell type were further normalized, scaled, clustered, and annotated using their specific markers, as described in supplementary table 2.

Detection of doublets and dead cells. We performed doublet identification using the DoubletFinder package and obtained the pANN score for each cell. Cells within pANN scores within the top 8.5% were identified as doublets (this number was calculated under the assumption that cells consisted of 15% doublets; however, doublets mixed by cells from one cluster could not be identified using the DoubletFinder method). Clusters with doublet rates above 40% were directly removed, and cells identified as doublets were also removed. These steps filtered out 1,597 cells, with 15,728 cells remaining. Doublets and dead cells were identified in subsequent annotation and analysis: Clusters with highly expressed mitochondrial genes were labeled as dead cells; Clusters that highly expressed markers of at least two cell types without any literature support for inter-transformation between these cell types were labeled as doublets. The dead cells and doublets were discarded after identification. After removing all low-quality cells we were left with 15,596 cells, of which 3,193, 4,512, 4,203, and 3,688 cells were obtained from days 0, 7, 14, and 21, respectively. These cells were considered high-quality and were used for all downstream analyses.

Enrichment analysis. After the markers of a certain group of cells had been determined, genes with top 200-fold change were fed into the "enrichR" package to find the most enriched term in the KEGG_2019_Mouse, GO_Biological_Process_2018 and GO_Molecular_Function_2019. P-values were calculated using a hypergeometric test and were adjusted using the false discovery rate (FDR) method. Terms with FDR less than or equal to 0.05 were considered significantly enriched. The positively and negatively enriched terms are listed in supplementary table 3 and 4.

2 Supplementary Figures and Tables

2.1 Supplementary Figures

Supplementary Figure 1. Pathological changes in mouse lung tissues during progression of bleomycin (BLM)-induced pulmonary fibrosis.

Supplementary Figure 2. **(A)**, Heatmap of top 20 marker gene expression in alveolar macrophages (top left), monocyte-derived macrophages (top right), and interstitial macrophages (bottom) in the progression of pulmonary fibrosis.

Supplementary Figure 3. **(A)**, Heatmap of the top 20 highly expressed genes for each AM cluster, representative marker genes are labeled. **(B)**, Bar plots showing the composition of AM clusters at different stages of pulmonary fibrosis. **(C)**, Gene expression of different chemokines, cytokines, and pro-fibrotic cytokines and for arachidonic acid metabolism in subclusters of AM and mo-Mac.

Supplementary Figure 4. **(A)** Bar plots showing the composition of mo-Mac clusters at various stages of pulmonary fibrosis. **(B)**, DDRTree Plot showing the trajectory development of mo-Mac. Three plots are colored by derived pseudotime (left), Seurat clustering results (middle), and sampling timepoints (right). **(C)**, Gene expression profiles of mo-Mac ordered according to pseudotime trajectory, with the x-axis representing pseudotime. Fibrosis-related genes are labeled.

Supplementary Figure 5. **(A)**, Heatmap showing all metabolic pathway activity of immune cell types at different stages of pulmonary fibrosis. Red indicates strong metabolic activity. **(B)**, Heatmap showing all metabolic pathway activity of different subclusters of macrophages at different stages of pulmonary fibrosis. **(C)**, Dot plot depicting the expression level of genes related to specific metabolic pathways in different immune cells and the percentage of cells expressing the gene at different stages of pulmonary fibrosis. **(D)**, Dot plot depicting the expression levels of genes related to specific metabolic pathways in macrophages and the percentage of cells expressing the gene at different stages of pulmonary fibrosis..

Supplementary Figure 6. **(A)**, UMAP plot depicting the reanalyzed immune cell clusters in lungs of human IPF patients. **(B)**, Heatmap of the canonical markers similarly enriched within mouse and human immune cell types. **(C)**, UMAP plot depicting the subclusters of macrophages in IPF patients. **(D)**, Heatmap of markers specifically enriched in different human macrophage subclusters.

Supplementary Figure 7. **(A)**, Heatmap showing the metabolic pathway activity of immune cell types from healthy donors and patients with IPF. Red indicates strong metabolic activity. Blue indicates weak metabolic activity. **(B)**, Heatmap showing the metabolic activity of different subclusters of human macrophages from healthy donors and patients with IPF.

2.2 Supplementary Tables

Supplementary Table 1. Marker genes of each cell clusters in mice.

Supplementary Table 2. Marker genes of each immune cell types in mice after annotation.

Supplementary Table 3. Enrichment result for AM clusters.

Supplementary Table 4. Enrichment result for mo-Mac clusters.

Supplementary Table 5. Marker genes of each cell clusters in human.

Supplementary Table 6. Marker genes of each immune cell types in human after annotation.

Supplementary Table 7. GSEA enrichment result and projection data.
