# Supplementary figures and images for: Dynamic atlas of immune cells reveals multiple functional features of macrophages associated with progression of pulmonary fibrosis
Source: Front Immunol. 2023 Sep 13;14:1230266. doi: 10.3389/fimmu.2023.1230266 (PMC10525351; doi:10.3389/fimmu.2023.1230266)

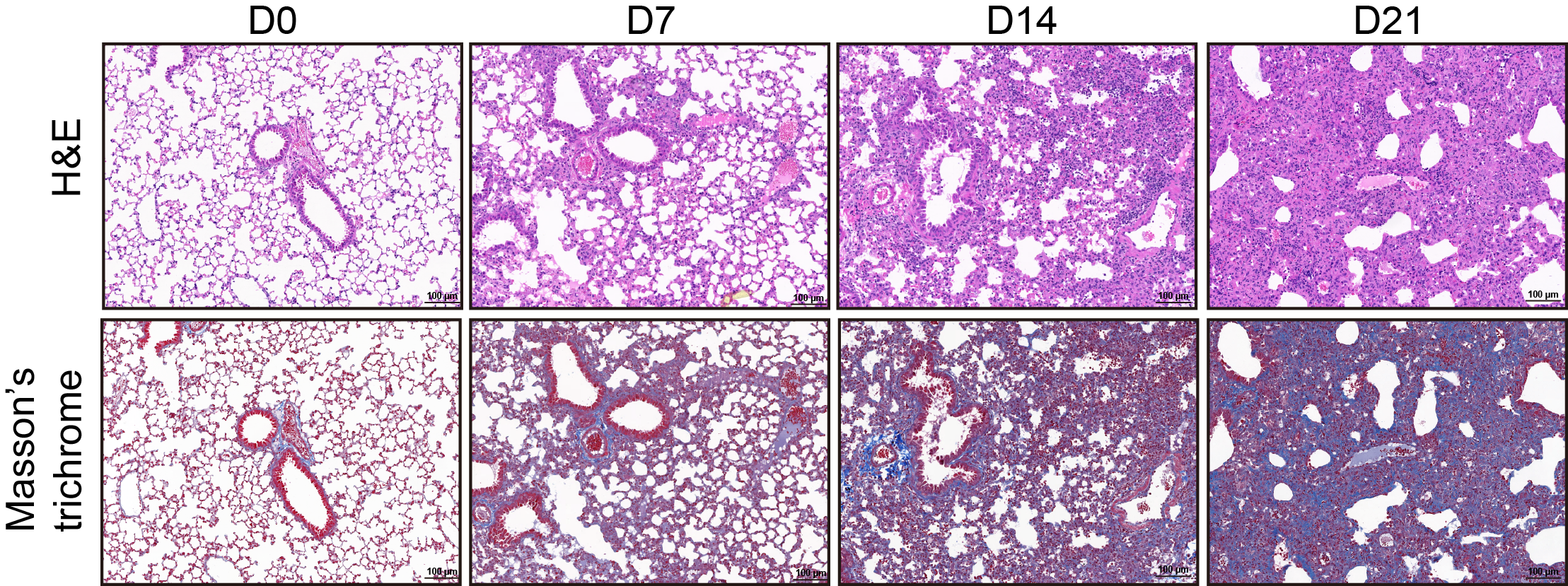

Supplement: Supplementary file 2 [file Image_1.tif]

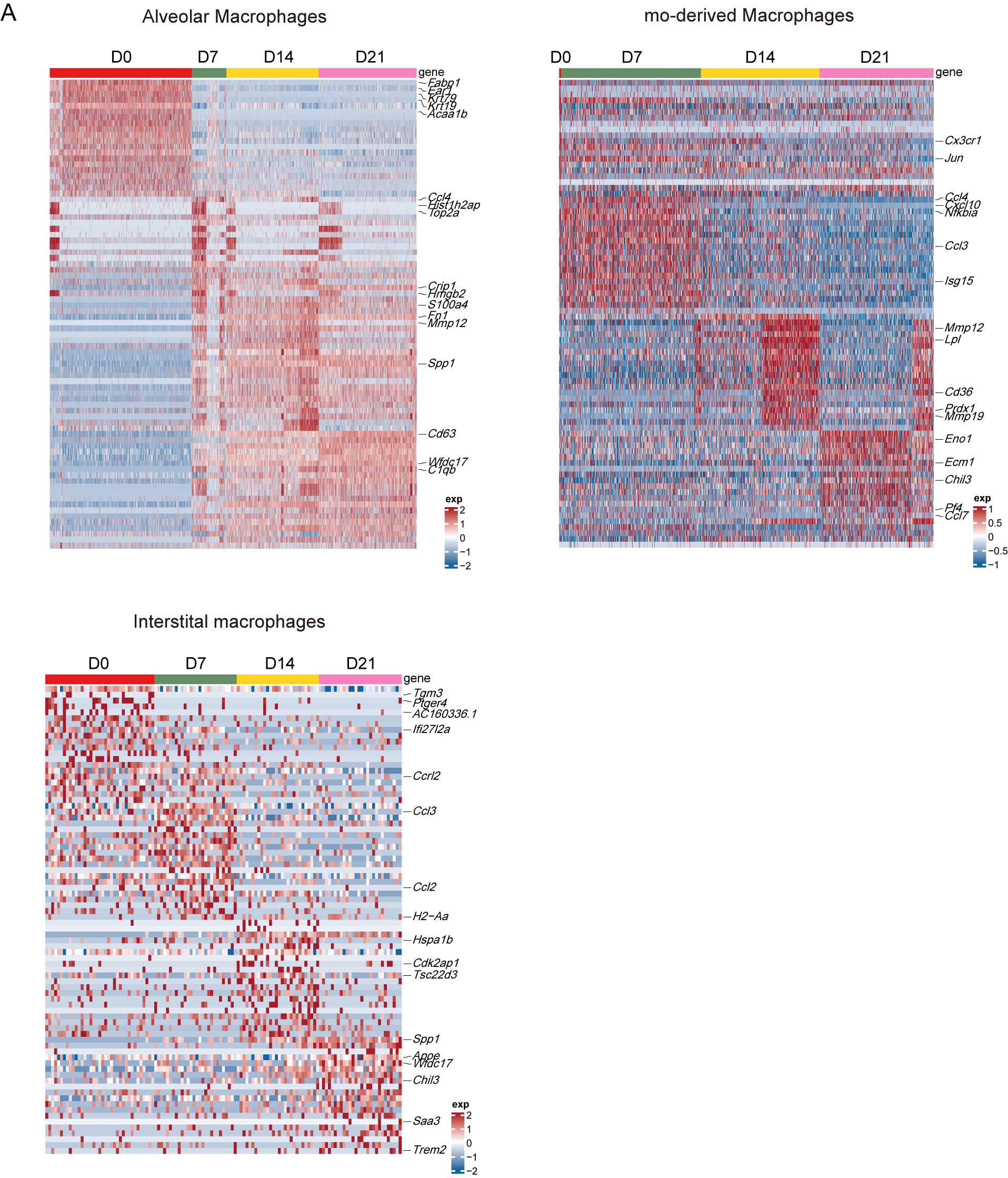

Supplement: Supplementary file 3 [file Image_2.tif]

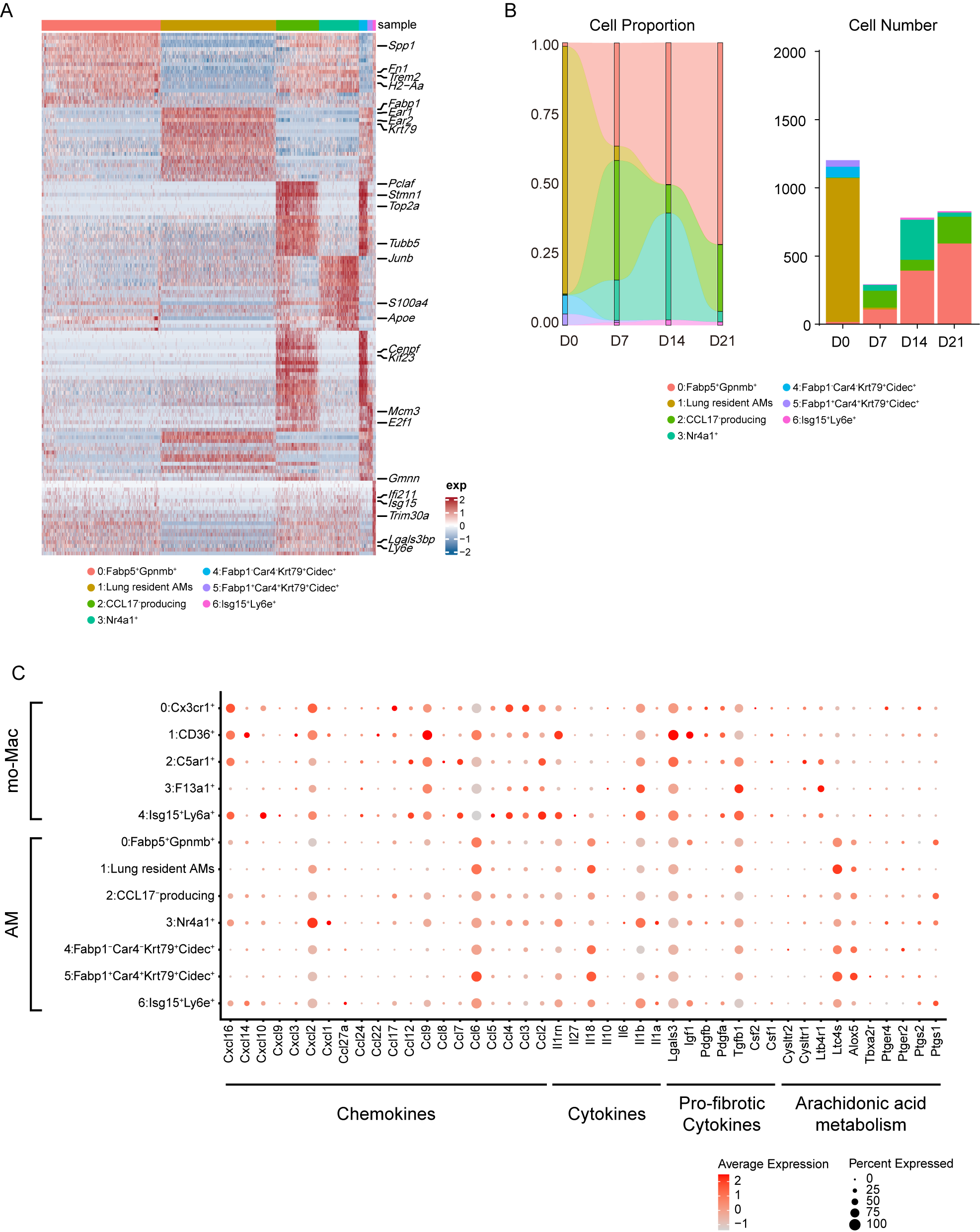

Supplement: Supplementary file 4 [file Image_3.tif]

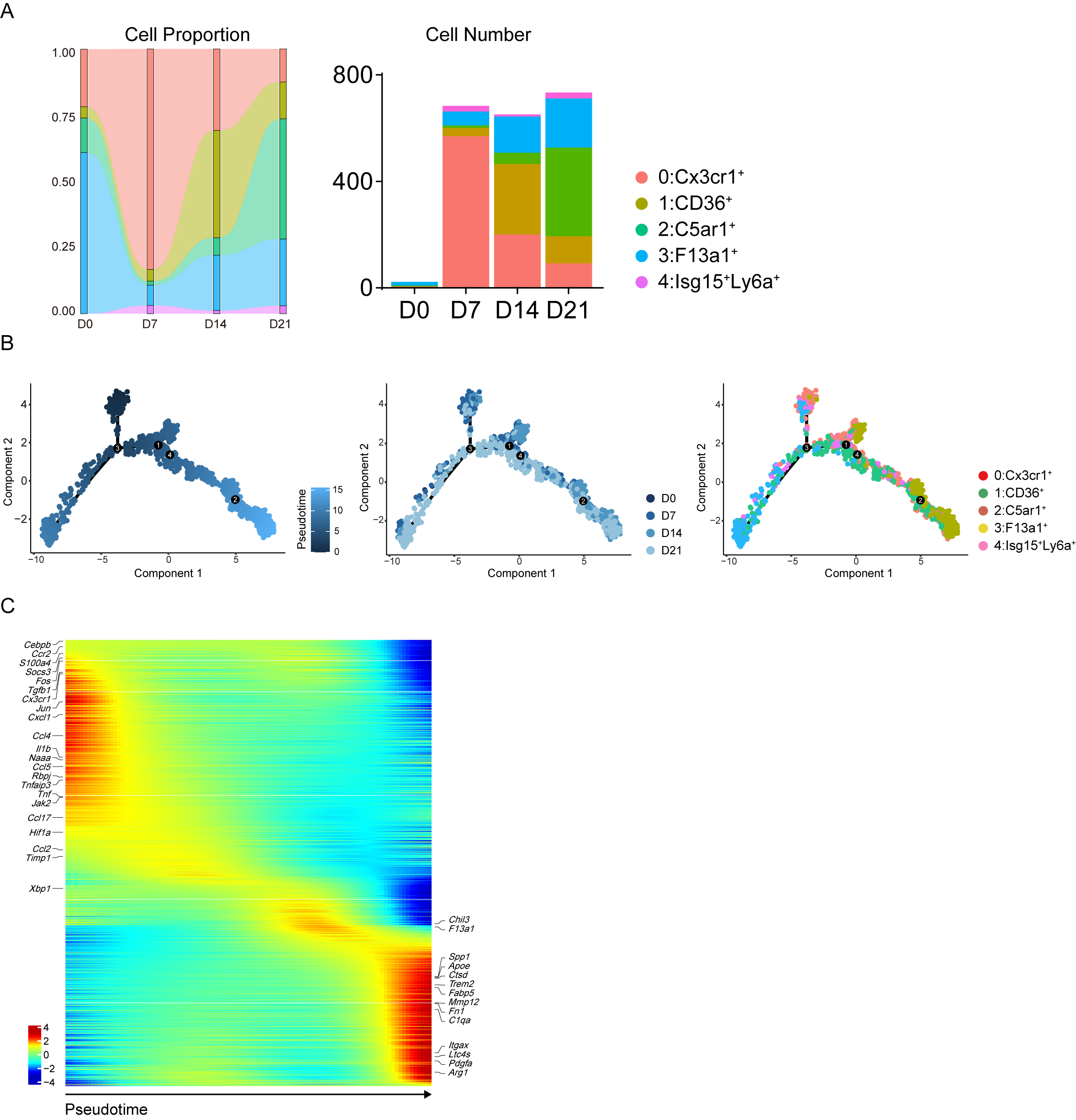

Supplement: Supplementary file 5 [file Image_4.tif]

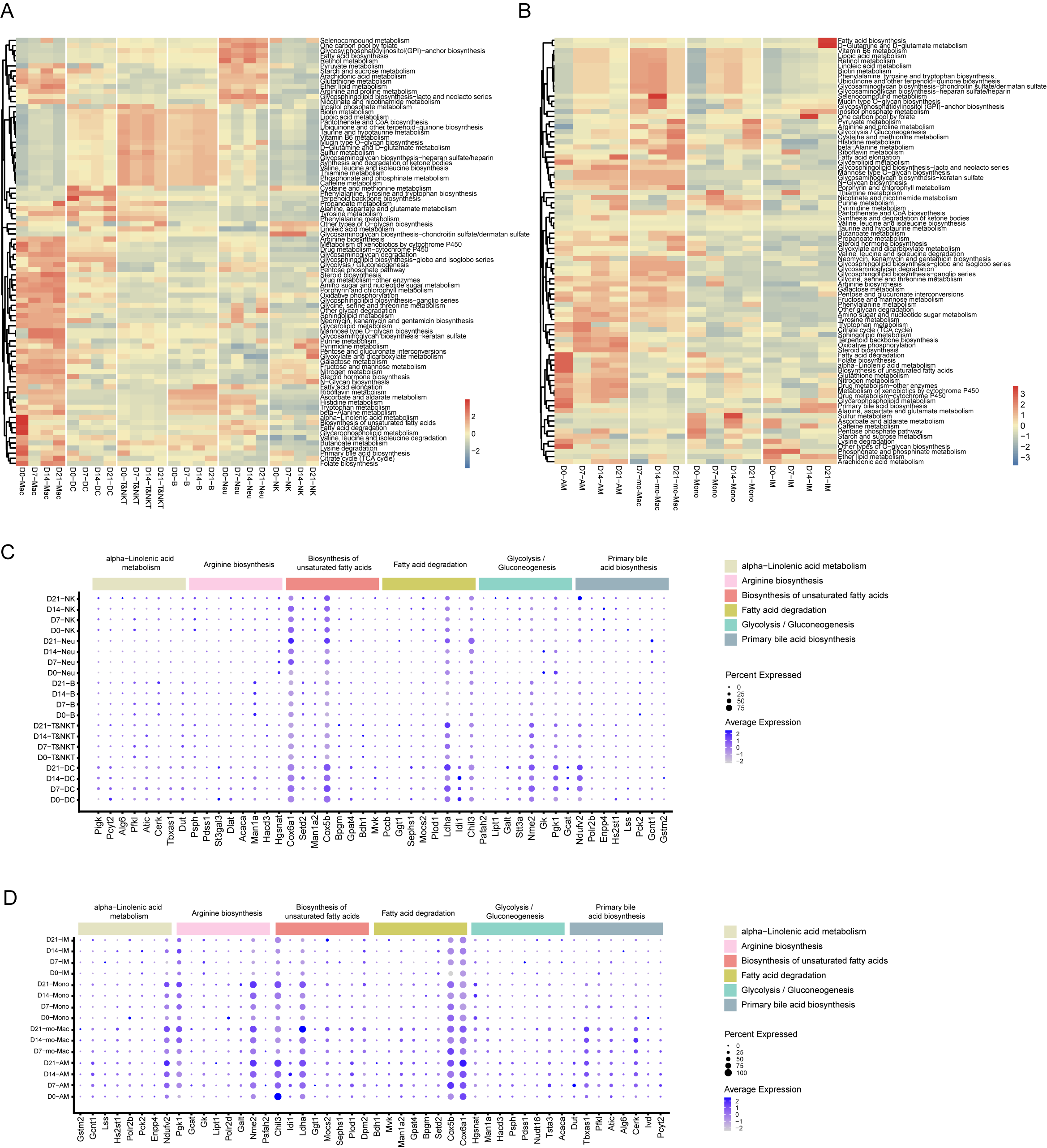

Supplement: Supplementary file 6 [file Image_5.tif]

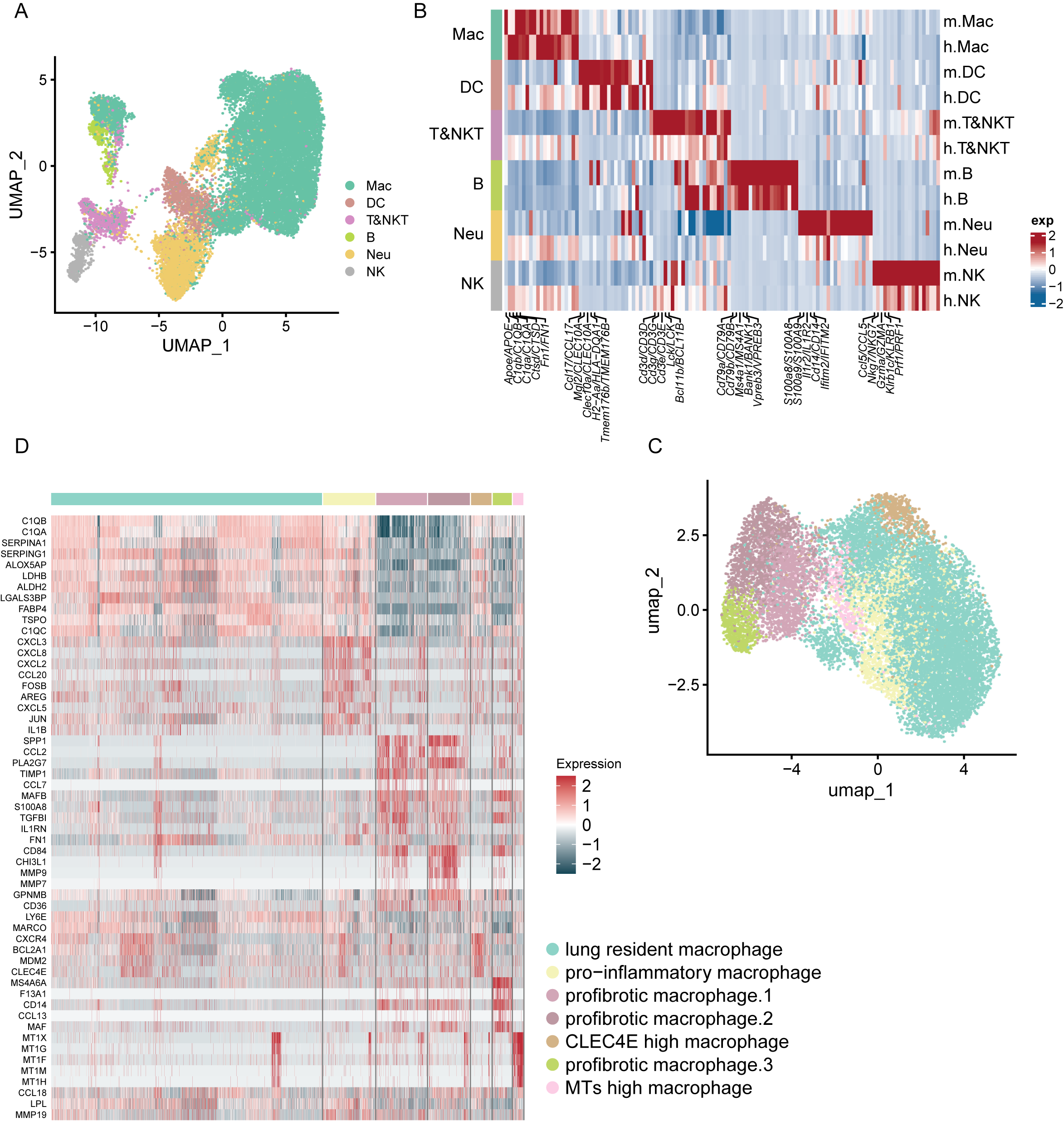

Supplement: Supplementary file 7 [file Image_6.tif]

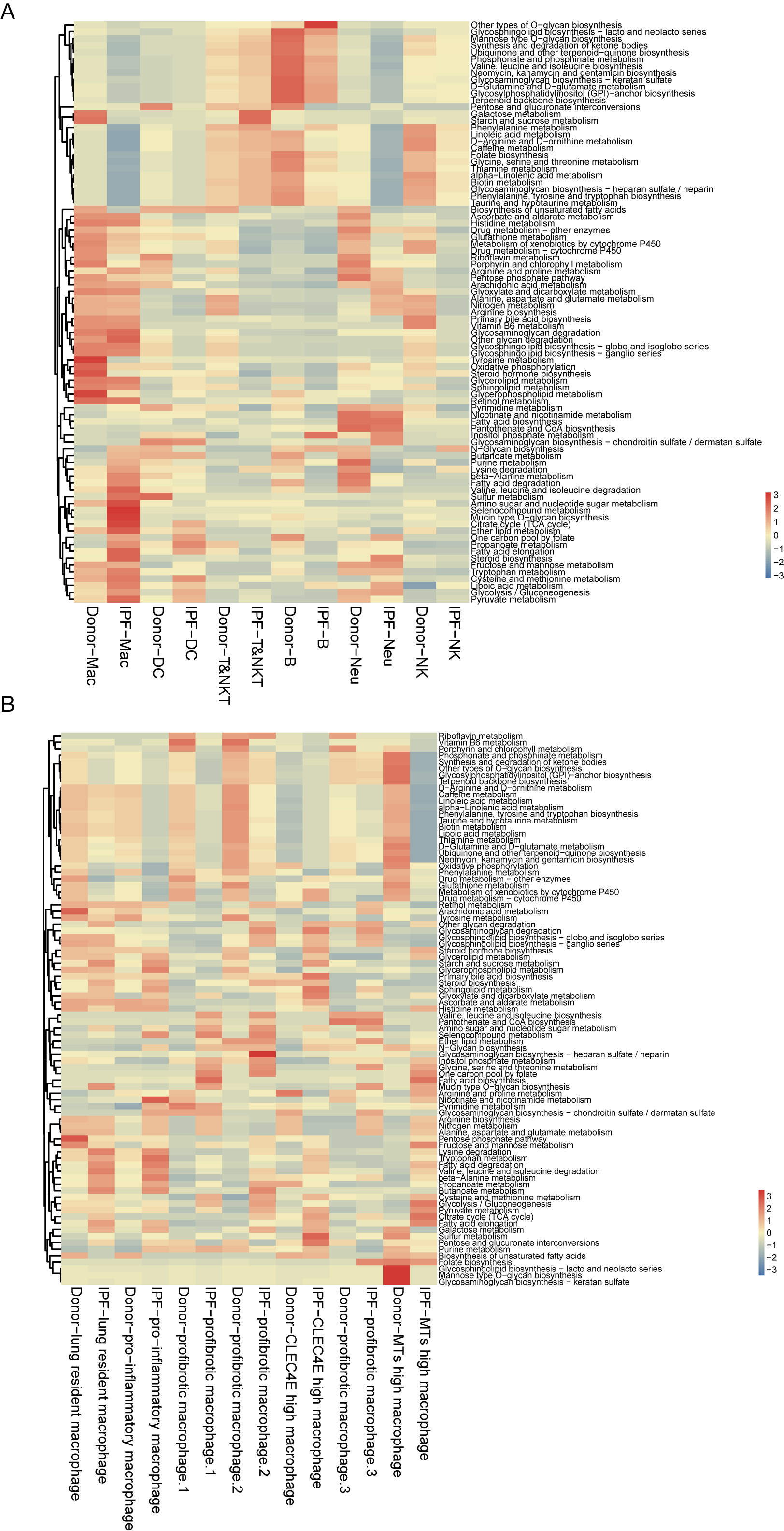

Supplement: Supplementary file 8 [file Image_7.tif]
